# Time to Be SHY? Some Comments on Sleep and Synaptic Homeostasis

**DOI:** 10.1155/2012/415250

**Published:** 2012-04-29

**Authors:** Giulio Tononi, Chiara Cirelli

**Affiliations:** Department of Psychiatry, University of Wisconsin, Madison 6001 Research Park Boulevard, Madison, WI 53719, USA

## Abstract

Sleep must serve an essential, universal function, one that offsets the risk of being disconnected from the environment. The synaptic homeostasis hypothesis (SHY) is an attempt to identify this essential function. Its core claim is that sleep is needed to reestablish synaptic homeostasis, which is challenged by the remarkable plasticity of the brain. In other words, sleep is “the price we pay for plasticity.” In this issue, M. G. Frank reviewed several aspects of the hypothesis and raised several issues. The comments below provide a brief summary of the motivations underlying SHY and clarify that SHY is a hypothesis not about specific mechanisms, but about a universal, essential function of sleep. This function is the preservation of synaptic homeostasis in the face of a systematic bias toward a net increase in synaptic strength—a challenge that is posed by learning during adult wake, and by massive synaptogenesis during development.

## 1. Introduction

In “Erasing synapses in sleep: is it time to be SHY?” (this issue), Marcos Frank provides an up-to-date evaluation of several aspects of the synaptic homeostasis hypothesis (SHY) of sleep function ([1, 2] and subsequent work). While this is not the place for a comprehensive discussion of the ideas and evidence behind SHY (Tononi and Cirelli, in preparation), Frank's commentary offers a welcome opportunity to address some of the experimental evidence about synaptic plasticity in wake and sleep, and to reconsider the involvement of additional factors affecting synaptic function, such as brain temperature and glucocorticoids. However, the way SHY is presented in the commentary suggests that it may be just as important to clarify what the hypothesis actually claims and what it does not. As acknowledged by Frank, SHY is eminently falsifiable, but one must make sure that what is put to the test are indeed SHY's tenets. With this in mind, it is useful to provide a brief summary of the motivations underlying SHY.

## 2. The Logic of SHY

Sleep is a behavior characterized by a reversible disconnection from the environment (when asleep we are “off-line”) and usually, but not always, by immobility. Sleep is present in all species studied so far (from fruit flies to humans), occurs from early development to old age, occupies a large fraction of the day, is tightly regulated (sleep homeostasis) and irresistible (it cannot be postponed indefinitely), and its loss leads to negative consequences, especially on cognitive functions [3]. These features strongly suggest that sleep must serve an essential, universal function, one that offsets the risk of being disconnected from the environment and the opportunity cost of not engaging in other behaviors. SHY is an attempt to identify this essential function. Its core claim is that sleep is needed to reestablish synaptic homeostasis, which is challenged by the remarkable plasticity of the brain. In other words, sleep is “the price we pay for plasticity [2].”

Briefly, the logic behind SHY is as follows. (i) The brain is extraordinarily plastic—changes in the number and efficacy of synapses, in intrinsic excitability, and in several other neuronal and glial parameters are the rule rather than the exception. Plasticity, of course, is essential for the development of neural circuitry and the adaptation to a changing environment. These noncontroversial premises are supported by overwhelming evidence. (ii) During wake, plastic changes are biased toward potentiation—for example, a net strengthening of synaptic efficacy and/or a net increase in the number of synapses. This is a novel claim, based on the premise that neurons usually signal important inputs by spiking more, rather than less. It follows that, to ensure that such signals percolate to other neurons deep inside the brain, connections conveying the signals should be strengthened, rather than weakened. A net increase in synaptic strength after wake is a key prediction of SHY and much effort has gone into testing it in different species using various experimental paradigms. (iii) A net increase in synaptic strength cannot be sustained indefinitely. This is because stronger synapses consume more energy, occupy more space, require more cellular supplies, saturate the capacity to learn and decrease signal-to-noise ratios (if more and more inputs are strengthened, neurons become progressively more excitable, and it becomes difficult to distinguish between signals that are important and ones that are not). (iv) Therefore, synaptic strength must be regulated and returned to a sustainable level, restoring synaptic homeostasis. In this way, the costs in terms of energy, space, supplies, signal-to-noise ratios, and learning capacity are restored to baseline. (v) Synaptic homeostasis is best achieved during sleep, a time when there is no demand for learning and neurons can sample most of their inputs in an unbiased manner through off-line spontaneous activity. By contrast, during wake neurons preferentially sample the particular subsets of inputs determined by interactions with the environment, and they are required to learn on-line. (vi) A similar need for synaptic homeostasis, hence for sleep, may exist during development. In many species, there is an initial overproduction of synapses, followed by net pruning down to adult levels [4]. Sleep would seem to be an ideal time for the selection of which synapses should remain and which should be pruned, through the unbiased, off-line sampling of a neuron's inputs. In summary, the core claim of SHY (and the reason it is called SHY) is that the universal, essential function of sleep is the restoration of synaptic homeostasis. If that turns out to be incorrect, so is SHY. For this reason, much effort has been devoted to evaluating structural, molecular, and physiological indices of synaptic efficacy before and after sleep. So far, evidence obtained using a variety of experimental approaches have been supportive in flies, rodents, and humans [5–14].

In addition to its core claim, SHY proposes some corollaries that are specific to animals showing slow wave activity (SWA) during sleep, such as mammals and birds. One corollary is based on the idea that synaptic number and strength influence the amplitude and slope of sleep slow waves. This is because stronger synapses increase neuronal synchrony, which in turn is reflected in larger and steeper slow waves in the EEG. Indeed, converging evidence indicates that the amplitude and slope of EEG slow waves is related to the number of neurons that enter an up state or a down state near-synchronously, and that synchrony is directly related to the number and strength of synaptic connections among them [15–17]. To the extent that this is correct, SHY entails, for example, that sleep SWA should be higher after wake and that it should decrease after sleep, in accord with evidence in many species of mammals and birds [18–21]. Moreover, SHY predicts that SWA should be locally regulated [1, 2]. For example, if a particular brain region undergoes a high amount of learning/synaptic potentiation in wake, that region should show a local increase in slow waves during subsequent sleep. This prediction has been confirmed by several studies, both in rodents and humans (e.g., [22, 23]).

A second corollary of SHY is that sleep slow waves may not simply reflect the number/strength of synapses, but they may be causally involved in synaptic homeostasis. Intriguingly, slow waves occur on average once a second or so, a frequency that is often associated with synaptic depression [12, 24–30]. The alternation of depolarized up states and hyperpolarized down states may also favor depression, and through spike-timing-dependent plasticity mechanisms, so would the increased synchrony caused by high synaptic strength [30, 31]. Finally, the neuromodulatory milieu in sleep, unlike that in wake, may also dampen potentiation and enhance depression [32, 33]. Irrespective of the particular mechanisms, the appealing feature underlying this corollary is that a positive link between synaptic strength and slow waves, coupled to a positive link between slow waves and synaptic depression, instantiates an elegant control mechanism that automatically regulates synaptic strength toward a baseline value [1, 2]. As shown through large-scale simulations [15, 31], the higher synaptic strength, the higher neuronal activity, and synchrony, yielding larger/steeper slow waves. On the other hand, the larger/steeper the slow waves, the more they produce synaptic depression. Moreover, when synaptic strength has been downregulated to a sustainable, baseline level, neurons are less synchronous, slow waves are small, and synaptic depression stops, avoiding the risk of run-away depression and possible memory loss. While this second corollary is certainly compatible with the mechanisms of synaptic depression mentioned above, as well as with some experimental findings involving manipulations of sleep SWA (e.g., [5, 34, 35]), so far the evidence supporting it remains limited and indirect.

## 3. Misunderstandings and Clarifications

This brief summary of the logic, core claim, and corollaries of SHY provides some context that should help, first, to clarify some misunderstandings that run through Frank's commentary and, second, to address some of the specific issues raised.

### 3.1. Function versus Mechanisms

The most important misunderstanding in Frank's commentary is the conflation between mechanisms and function. As briefly outlined above, SHY is first and foremost a hypothesis about the universal, essential function of sleep, not about which specific mechanisms mediate that function. From the start, SHY assumed that the proposed function of sleep—synaptic homeostasis—might be carried out through different mechanisms in brain structures with different sleep rhythms, such as the hippocampus, and in species with a very different brain, such as *Drosophila* [1, 2]. How exactly *Drosophila* neurons might achieve synaptic homeostasis is an interesting mechanistic question—as pointed out by Frank, *Drosophila* neurons may not undergo slow oscillations—but it has no bearing on whether the core claim of SHY is true or false. Given that *Drosophila* does sleep [36, 37], the central issue for SHY is whether its neurons need synaptic homeostasis, and whether synaptic homeostasis requires sleep. So far, the evidence is positive [7, 8, 10, 11].

The focus on mechanisms rather than function may explain why, on several occasions, Frank is troubled by the perceived “vagueness” of SHY concerning the particular molecular pathways that may underlie synaptic homeostasis. In fact, SHY is purposely liberal about specific mechanisms not out of vagueness, ignorance (though still substantial), or a desire to eschew falsification, but because it is the proposed function of sleep that is universal, not the particular mechanisms, especially in view of the extraordinary variety of cellular and molecular pathways involved in plasticity. Indeed, if synaptic homeostasis turns out to be implemented differently in different species and brain structures, the hypothesis would be strengthened, not weakened: as with convergent evolution, if the same function is achieved with different means in different species, that function is probably fundamental.

### 3.2. Synaptic Homeostasis versus Activity-Dependent Homeostatic Plasticity

A second misunderstanding is closely related to the first: Frank often portrays SHY as if it were a hypothesis about sleep implementing a particular mechanism, *synaptic scaling*, first observed *in vitro* after massive manipulations of neuronal activity [38], rather than a core functional effect, the “generalized depression or downscaling of synapses” [1]. In other words, Frank equates synaptic homeostasis—the proposed function of sleep—with a specific mechanism of activity-dependent homeostatic plasticity. Homeostatic plasticity refers to an array of phenomena whose goal is to maintain a key parameter, neuronal activity, around some set-point value [38]. Synaptic scaling is the best characterized mechanism underlying homeostatic plasticity and, as Frank discusses at length, it allows neurons to counteract excessive or insufficient activity by down- or up-scaling all their synapses by the same factor [38]. Homeostatic plasticity is typically contrasted with synapse-specific, associative “Hebbian” plasticity both conceptually and in terms of the molecular mechanisms involved, although experimentally the distinction has become more complicated and nuanced [38]. SHY noted that scaling principles of the kind observed with homeostatic plasticity might be involved in synaptic homeostasis during sleep. Indeed, scaling is an attractive mechanism because it can produce a net reduction in synaptic strength while preserving the relative strength of synapses. On the other hand, SHY clearly stated that the primary variable regulated by synaptic homeostasis is synaptic strength, rather than the average neuronal firing rate, as in homeostatic plasticity [38]. Moreover, other mechanisms, including activity-dependent long-term depression, are compatible with SHY as long as depression is generalized to the majority of synapses, thanks to the unbiased off-line activity of sleep. For example, in large-scale simulations, synaptic homeostasis was implemented through generalized synapse-specific depression, with the amount of depression inversely proportional to synaptic strength [31]. In this way, signal-to-noise ratios increased and performance improved. Furthermore, SHY explicitly considered the possibility that some synapses may be strengthened during sleep [2], thereby further enhancing competition, as long as the net effect was an overall reduction of synaptic efficacy. Finally, SHY emphasized that the specific mechanisms involved in synaptic homeostasis can vary—borrowing from homeostatic plasticity, long-term depression, depotentiation, and so on—as long as the end result was a net depression of synapses: “Whichever the specific mechanism, the hypothesis is that a generalized synaptic downscaling during sleep, including possibly the downselection or pruning of certain synapses, serves to ensure the maintenance of balanced synaptic input to cortical neurons” [1, 2].

Retrospectively, the conflation of “synaptic homeostasis” and “homeostatic plasticity” may be attributed to the confusion generated by the shared concept of “homeostasis.” SHY pointedly refers to homeostasis to emphasize that sleep serves a fundamental regulatory function—maintain an appropriate level of a key biological parameter, namely, synaptic strength—in the face of variations imposed by learning and development. Homeostatic plasticity is called so because global synaptic scaling is used to regulate another biologically relevant parameter—the level of neuronal activity [38]. Considering that scaling mechanisms involved in homeostatic plasticity may also be involved in maintaining synaptic strength around stable levels, and that activity and plasticity are linked, a certain amount of confusion was perhaps inevitable. This confusion was compounded because SHY loosely referred to the postulated net decrease in synaptic strength as “downscaling.” However, SHY never necessarily implied either precise proportionality (all synapses scaled down by the same factor) or a specific molecular mechanism. For this reason, later publications have employed the more neutral term synaptic “renormalization” to describe how synaptic homeostasis is reestablished [6–9, 39, 40].

### 3.3. Wake and Long-Term Potentiation (LTP)

In a similar vein, SHY does not assert that plasticity during wake should be exclusively equated with homosynaptic, associative, “Hebbian” long-term potentiation. Again, the core claim is that wake is associated with a net increase in synaptic strength, irrespective of the particular mechanisms involved, and notwithstanding the possibility that synaptic depression may also occur [1, 2]. As briefly explained above, the prediction that learning during wake should lead to a net increase in synaptic strength is based on the idea that neurons should signal important events to the rest of the brain through increased rather than reduced firing, implying that learning, too, should be biased toward potentiation. For this reason, the most important evidence for SHY is the demonstration, using structural, electrophysiological, and molecular tools in different species, that net synaptic strength increases with wake and decreases with sleep. By contrast, much of the evidence that Frank considers problematic for SHY has to do with instances in which some molecules that may be implicated, say, in synaptic depression, may occasionally be highly expressed in wake; or in which a particular molecule that is highly expressed in wake, say BDNF or Arc, can be involved in both potentiation and depression. Once again, given the extraordinary complexity of plasticity mechanisms, the large number of possible molecular and electrophysiological interactions, the differences between brain structures and species, and the complicated influence of neuromodulators, one would not expect a simple mapping between synaptic homeostasis and particular molecular or electrophysiological mechanisms. Thus, when Frank argues that the “simplistic” idea that wake and sleep are dominated by net synaptic strengthening and weakening, respectively, is based on a “very narrow view of brain plasticity” (which is certainly multifarious) or that SHY is “oddly disconnected from our rapidly evolving view of synaptic plasticity” (which is becoming increasingly complex), he has it exactly backwards. The appeal of SHY is precisely that it proposes a universal function for sleep—synaptic homeostasis—in the face of the variety and complexity of plasticity mechanisms across different brain circuits, species, developmental phases, and behavioral contexts. What matters for function is the end result, irrespective of the particular molecular interactions involved, and the particular role of specific molecules. SHY predicts that wake will result in a net increase in synaptic strength, and that sleep is needed for its renormalization. If the data show eventually that such net changes do not occur, SHY is wrong. But if such net changes do occur, they most likely involve multiple, complicated, and interacting mechanisms, and different ones in different species and brain structures.

### 3.4. Cellular Consequences of Synaptic Homeostasis

Finally, in his commentary, Frank restricts his discussion of the potential benefits of synaptic homeostasis to the increase in signal-to-noise ratios, which would help memory consolidation. SHY certainly proposes that sleep-dependent synaptic renormalization should increase signal-to-noise ratios and thereby enhance performance, as suggested both by computational and experimental work [22, 31, 35, 41]. However, SHY has always ascribed to sleep-dependent synaptic homeostasis a much broader function in counteracting the accumulation of synaptic strength [1, 2]. In addition to a reduction in signal-to-noise ratios, high synaptic strength has other costs, including higher energy consumption (synaptic signaling accounts for most of the brain's energy, and stronger synapses consume more energy [42, 43]); decreased space available for further growth (stronger synapses are usually also larger [44]); increased need of cellular supplies, because synaptic plasticity enhances the turnover of proteins and various cellular constituents, requiring a substantial involvement of transport processes, energy delivery processes, and endoplasmic reticulum functions including protein folding [45–48]. Moreover, a net increase in synaptic strength may lead to saturation of the capacity to learn, which can occur quite rapidly in cortex and hippocampus [49–53]. For example, a recent experiment found that synaptic potentiation by direct electrical stimulation of the cortex was difficult to induce after wake but easy after sleep, again suggesting that several hours of wake are enough to bring cortical synapses close to their level of saturation [5]. In short, the benefit that sleep provides for memory consolidation, while important, is certainly not the sole reason underlying the need for synaptic homeostasis.

## 4. Specific Issues

Having clarified the core claim and corollaries of SHY, it is helpful to consider specific issues raised by Frank's commentary one-by-one. Some of these issues offer excellent points of discussion, highlight areas of current ignorance, and suggest relevant experiments for the future.

### 4.1. On the Mechanisms of Plasticity in Sleep

*“(SHY) argues that learning is largely mediated by LTP. …Learning is a deceptively simple term for a complex set of neural events … while some forms of learning may be associated with LTP, others are not or involve a mixture of LTP and LTD-like synaptic changes … The potentiation hypothesized to occur in wakefulness is considered Hebbian …*”

SHY claims that learning—an enduring modification of brain circuits as a result of perception, cognition, and action that occurs throughout wakefulness, is inherently biased towards a net increase in synaptic strength. The postulated net increase in synaptic strength after wake was termed “LTP-like” with reference to the most studied experimental paradigms for producing long increases in synaptic efficacy, to indicate that it was an increase (potentiation) and that it was enduring (long term). In reviewing the available evidence for or against SHY when the hypothesis was first proposed, it was pointed out that there were many correlative, indirect data, such as gene expression changes, which were consistent with a predominance of LTP-like changes during wake, and moreover that LTP-like changes underlie the majority of learning paradigms that have been studied from a cellular perspective. This was before direct experimental tests inspired by the hypothesis could be performed, including the demonstration of wake-associated increases in evoked responses, in miniature synaptic potentials, in AMPA receptor density at the synapse, and in the number of synapses themselves [5–14].

As always, however, SHY did not endorse a particular mechanism of plasticity (hence LTP-like), only the end result. It never stated that wake-related potentiation is accounted for by a single mechanism such as classic “Hebbian” homosynaptic plasticity (occurring only at the stimulated synapse), and in fact terms such as “Hebbian” or “homosynaptic” were never mentioned [1, 2]. Indeed, it is fair to ask what “homosynaptic” truly means, in light of the current evidence for synaptic tagging and capture, and recent data showing that the primary functional unit for long-term synaptic potentiation may be a dendritic branch, not an individual synapse [54].

Similarly, SHY never claimed that learning during wake only occurs via synaptic potentiation—just that the overall net result is biased towards potentiation. Indeed, there are several well-characterized forms of learning “by depression” that certainly occur during wake. These include reversal learning in the hippocampus, fear extinction in the amygdala, familiarity recognition in perirhynal cortex, and other forms of “behavioral flexibility” that involve either decreasing the response to a familiar stimulus or forgetting old strategies, objects, or spaces [55]. Fittingly, it appears that enduring synaptic depression is associated more with forgetting what was previously known, than with acquiring new knowledge. Consistent with this notion, acute stress impairs hippocampus-dependent memory retrieval, and hippocampal synaptic depression seems to play a role in this effect [55].

Of note, Frank quotes the work of Manahan-Vaughan and colleagues to make the point that learning includes both synaptic potentiation and depression. He fails to mention, however, that these authors, in discussing the overall implications of their results, noticed that exposure to a novel environment induces synaptic strengthening in all the four types of hippocampal synapses studied, while synaptic depression was not as universal. In their most recent paper, Manahan-Vaughan and colleagues concluded that synaptic potentiation “may represent a fundamental coding response to changes to the environment,” whereas synaptic depression may add “a more qualitative component” ([56] page 2446 and Figure 10). Also, a study quoted by Frank in the section “Learning and LTP” as evidence for depression [57] is actually noncommittal about the mechanisms of learning but rather shows that long-term consolidation of spatial memory, 24 hours after learning, may require synaptic depression.

As anybody studying brain mechanisms of plasticity knows all too well, many forms of LTP-like paradigms have been described in different species, brain structures, and developmental times. Controversies have raged as to whether classic LTP/LTD paradigms induce changes resembling those occurring physiologically, whether changes are primarily postsynaptic, presynaptic, or both; whether changes are strictly “Hebbian” or not; whether *in vivo* synaptic changes are driven by mean firing rates, by spike-timing-dependent plasticity, or some other combination of mechanisms; whether changes are strictly confined to individual synapses or to a larger volume of neuropil; as to the involvement of glia, the role of neuromodulators, the participation of mitochondria and energy constraints, and of course to the particular molecular pathways and scores of molecular mechanisms that seem to be involved under different conditions. In short, the mechanisms of learning and plasticity are extraordinarily complex, involving at least dozens of different synaptic mechanisms and hundreds if not thousands of molecules, many still unknown or incompletely understood and run the gamut from short-term, medium-term, and long-term potentiation, to depression, depotentiation, spike-timing-dependent plasticity, scaling, intrinsic plasticity, structural plasticity, metaplasticity, and so on. SHY fully acknowledges this extreme biological complexity, but proposes that, (literally) at the end of the day, energy and information constraints on the brain—the fact that strong firing must be reserved for important signals that need to percolate among long chains of neurons—necessarily bias learning toward increasing overall synaptic strength.

*“… surprisingly, many … findings cited in support of SHY are inconsistent with net synaptic downscaling … ”*

Again, one needs to distinguish between the end result and the specific mechanism. As already stated, the evidence for a net decrease in synaptic strength or number after sleep is strong. If this were not the case, SHY would have to be abandoned. On the other hand, just as SHY did not commit to a specific mechanism resulting in net potentiation after wake, it also did not endorse a particular mechanism for bringing about net synaptic depression during sleep. While SHY referred to the recently discovered mechanisms of global synaptic scaling as a possible means for proportionally depressing synapses without compromising their relative strength, it did not endorse the specific mechanism. Indeed, an earlier paper already pointed out in 2001, as Frank does now in his commentary, that the available evidence was not supportive: “BDNF, which plays a critical role in synaptic scaling *in vitro*, is expressed at higher levels in the waking rather than in the sleeping brain” [58]. Moreover, SHY pointed out explicitly that synaptic scaling was meant to “ensure that neurons maintain a regulated firing level in the face of uncontrollable changes in their input,” whereas synaptic homeostasis in sleep was meant to “ensure primarily the homeostatic control of synaptic weight, and only indirectly of neuronal firing levels” ([2], page 53). This point was further addressed in 2009, in a study that showed that cortical firing rates increase in the course of wake and decrease during sleep [17]. Since these sleep/wake changes in cortical firing are small, of the order of a few Hz, it was deemed unlikely that they could trigger the same homeostatic changes observed by Turrigiano and colleagues after extreme changes in firing rates ([17], page 874).

*“What seems more likely is that sleep is characterized by multiple forms of synaptic plasticity, including classic Hebbian LTP and LTD, as well as downscaling and upscaling. This may explain why the evidence for “net” downscaling after sleep critically depends on what is measured (*e.g.,* neuromodulin versus BDNF)  …” *

Once more, it is virtually certain that multiple forms of plasticity can occur both in wake and in sleep—what matter for SHY is only whether wake inevitably tends toward net potentiation, and sleep is needed to restore homeostasis. Before more direct tests of net synaptic strength could be performed, the overall picture provided by gene expression studies in the waking and sleeping brain [59–61], though imperfect and indirect, was at least broadly compatible with the core claim of SHY. However, it is clear that the expression level of any single molecule, be it BDNF, Arc, Homer, neuromodulin, or any other, is not substitute for a direct assessment of net synaptic strength. This is indeed what needs to be measured, and this is why, after SHY was first proposed, many experiments were performed to try and assess synaptic strength directly, using as many experimental approaches as possible: first molecular and electrophysiological markers *in vivo* (AMPA receptors density in synaptoneurosomes and slope of evoked responses), then electrophysiological markers *ex vivo* (minis), and finally structural markers (synapse size and number).

Incidentally, while as Frank suggests the results of early studies of gene expression in wake and sleep may have inspired the idea of a bias toward potentiation in wake, the central motivation for SHY was the search for a function for sleep that should be carried out off-line rather than on-line, and one that could have universal significance. Many ideas about the function of sleep start from the notion that wake may result in the accumulation of some “toxin” and that sleep may be necessary to restore the brain to a healthier state. If one adds the insight that one needs to explain the apparent need for such restoration to occur off-line, despite the considerable risk imposed by the disconnection from the environment, one could say that the “toxin” that necessarily accumulates in wake may be synaptic strength itself.

*“… the term “net” is somewhat nebulous …”* *… “This broad description of the mechanisms of downscaling has the advantage that any evidence of synaptic weakening after sleep … can be cited in support of the theory. It is disadvantageous in that no single, clear mechanism is presented for careful and in depth investigation… One important future direction is to delve more deeply into the underlying mechanisms of SHY. To date, this has received less attention than studies aimed at collecting supportive findings.”*

After evaluating the evidence for SHY as if the hypothesis were not about function but about the occurrence during sleep of a specific mechanism—activity-dependent scaling—and finding the evidence wanting, Frank then remarkably goes on to chastise SHY because it does not single out a single, clear mechanism. Once more, Frank seems to value whatever SHY may or may not say about mechanism much more than what it says about function. SHY aims at identifying a universal, essential function for sleep—a function that must necessarily transcend specific mechanisms if it is to apply to many species, brain circuits, and developmental periods in the face of an extraordinary biological diversity. This does not mean, however, that SHY ignores mechanisms. A case in point is the suggestion that, in mammals and birds, sleep slow waves may constitute an advantageous mechanism both for sampling in an unbiased manner the overall synaptic strength impinging on a neuron, and for renormalizing it in a controlled, self-limiting way [1, 2]. But of course, whether and how, exactly, slow waves may do so (with or without the contribution of other features of sleep, such as spindles), whether synaptic strength decreases in a proportional manner or enforces a competition between stronger and weaker synapses, older and newer memories, and so on, are questions that are as important as they are difficult to address experimentally.

Incidentally, characterizing the experimental work conducted so far as aimed at “collecting supportive findings” is puzzling because it suggests that evidence was collected selectively. The core claim of SHY, according to which wake led to a net increase in synaptic strength and sleep to a net decrease, had never been considered or tested before. *A priori*, the results of many different experiments conducted in different species and with different approaches could easily have been negative—most of the plasticity literature implicitly assumes that a balance between potentiation and depression is a given—or it might have turned out that sleep leads to a net potentiation. Collecting evidence that turned out to be supportive (so far) is not the same as “collecting supportive evidence.”

### 4.2. On the Role of Slow Wave Activity in Synaptic Weakening

Sleep SWA features in two prominent corollaries of SHY, in the first as a possible sensor of synaptic weight, and in the second as a possible effector of sleep-dependent synaptic renormalization. Frank discusses these two corollaries at some length, and it is important to maintain a clear distinction between these two postulated roles for sleep SWA.

The evidence for SWA as an index of synaptic strength rests on the fact that the sleep slow waves recorded from the scalp by the EEG are a reflection of near-synchronous transitions between up and down states in large populations of cortical neurons [62, 63]. Both theoretical considerations [64], large-scale simulations [15] and empirical studies, [16, 17] indicate that the amplitude and slope of sleep slow waves are related to the number of neurons that enter an up state or a down state near-synchronously, and that synchrony is directly related to the number and strength of synaptic connections among them. More specifically, the data showing that SWA can be used as a “proxy” of synaptic strength come from studies in humans using high-density EEG and, in animals, from experimental approaches that can reveal the local aspect of sleep regulation. For instance, in humans, SWA increases locally over parietal cortex following learning of a visuomotor task [22], while arm immobilization during the day, which leads to a decrease in motor performance and sensory evoked responses, consistent with synaptic depression, is followed by reduced SWA over the contralateral sensorimotor cortex [65]. Cortical potentiation and depression triggered in humans by paired-associative stimulation also result in increase and decrease in SWA, respectively [66]. In rats, training on a reaching task known to induce long-term synaptic potentiation results in a local increase in SWA in the activated motor region [23]. Cortical infusion of BDNF, whose local brain application *in vivo* is sufficient to induce synaptic potentiation, results in an increase in SWA only in the injected cortex [67]. Moreover, in the rat cortex, the wake-related increase in the slope of local field potentials discussed above correlates with the increase in SWA: the steeper the slope at the end of wake, the higher SWA at sleep onset [5].

There are also developmental studies that link SWA to synaptic strength. In both mice and cats, visual deprivation during the critical period, which is associated with synaptic depression [68], results in a 40% decrease in SWA [69]. Moreover, recent and growing evidence in humans suggests that the well-documented inverted U curve of SWA during development, with an early progressive increase during childhood, followed by a rapid decline, may reflect the equally well-documented early cortical increase in synaptic density, followed by synaptic pruning during adolescence [70–74]. In summary, it seems that the evidence for SWA as a sensor of synaptic strength is quite strong. Instead, the role of SWA as an effector of sleep-related synaptic renormalization remains hypothetical: SWA may not be an effector at all or may be just one of the mechanisms to achieve synaptic downregulation, and perhaps only in some animal species [2, 6, 8, 31].

*“if decreases in SWA directly reflect decreases in synaptic strength … … physiological markers of synaptic weakening should be detectable when SWA first declines*….*The few studies*….*have produced very mixed results*….*”*

Frank rightly notices that more studies are needed to show that the time course of synaptic renormalization is linked to that of sleep. However, some evidence that markers of synaptic strength decline in proportion to sleep already exists. In flies, spine pruning after an enriched experience occurs only if they are allowed to sleep, but not if they remain awake [8]. Crucially, spine density was negatively correlated with the amount of sleep during the last 7 hours, as well as with the maximal duration of sleep bouts [8]. Turning to electrophysiological markers in rodents, local field potentials were recorded from left frontal cortex after electrical stimulation of the right frontal cortex. After transcallosal stimulation, the slope of the first negative component of cortical evoked responses—a monosynaptic response—increased after wake and decreased after sleep [5]. Importantly, changes in slope were correlated with the duration of prior wake or NREM sleep. In relation to this study, Frank points out a “discrepancy” with another study in the visual cortex, in which evoked responses declined in amplitude during the active phase [75]. In fact, the two studies were designed to ask very different questions, and thus differed in a crucial experimental detail. The Vyazovskiy study was designed to assess how sleep/wake history affects cortical strength, and, therefore, the behavioral state was kept constant (quiet wake) at the time the evoked responses were collected. By contrast, Tsanov and colleagues compared responses collected during sleep with those collected during wake. This is crucial because independent of sleep/wake history, evoked responses are much larger during sleep than during wake. Thus, inferences about 24-hour changes in cortical strength are simply impossible to make if one does not control for behavioral state. A recent study in humans also found that human cortical evoked responses, reflected in the immediate (0–20 ms) electroencephalographic reaction to transcranial magnetic stimulation, progressively increased with time awake, from morning to evening and after one night of sleep deprivation, and decreased after recovery sleep [14]. This study, as the rat study, collected evoked responses during the same behavioral state (wake), and after controlling for drowsiness [14].

### 4.3. On the Evidence for SHY, in Mammals and in Insects

*“… SHY is supported by an impressive number of findings … mostly reported by the same group …”*

Over the past several years, the core claim of SHY has been put to direct test using several experimental approaches aimed at estimating synaptic efficacy, in different species, *in vivo* as well as *ex vivo*. These include molecular studies in rats (changes in AMPA receptors; [5]) and flies [7]; electrophysiological studies in rodents and humans, including changes in the slope of evoked responses *in vivo* [5], changes in cortical excitability as assessed by transcranial magnetic stimulation [14], and changes in frequency and amplitude of minis *in vitro* [6]; as well as morphological studies showing changes in the number of synapses in flies [7, 8] and mice [9]. None of these approaches, taken in isolation, can offer an exhaustive, unambiguous view of synaptic efficacy: morphological changes in the number or size of synapses are not necessarily accompanied by changes in their efficacy; changes in the number of AMPA receptors in synaptic fractions cannot tell how functional those receptors may be; changes in spontaneous miniature synaptic potentials (minis) measured *ex vivo* may not accurately reflect the efficacy of synapses when neural activity is high *in vivo*; and changes in field evoked responses after electrical or magnetic stimulation cannot easily distinguish between changes in synaptic strength and changes in neuronal excitability due to other causes. Nevertheless, taken together, these various sources of evidence complement each other. While it is true that the results mentioned above were obtained from the same laboratory (or through collaborations), several of the findings have already received independent support from three different laboratories [10–13]. Obviously, further direct tests of the main tenets of SHY—in different species, brain structures, and developmental periods—will be important to establish if and to what extent the predictions of the hypothesis can be generalized.

*“… the most dramatic evidence of SHY is found in ectothermic insects …”*

Frank sees the structural changes in flies as the most dramatic examples of sleep/wake effects on synaptic strength. While this may indeed be the case, trying to compare effect size across studies done in different species and using very different methods is tricky. At this point, it is not obvious that when comparing wake to sleep, the 100% increase in the frequency of miniature postsynaptic currents observed in rodent cortex [6], or the 30% increase in the number of synaptic AMPA receptors across the entire rat cortex [5], reflects a less significant change in synaptic strength than the 2-fold increase in size of presynaptic terminals or the 30% increase in spine density seen in the fly brain [8]. What matters most, of course, is that all of these findings go in the same direction.

*“It also appears that SWA cannot be a common mechanism for downscaling in mammals and insects.”*

It is currently unknown whether neurons in the fly brain (or in the brain of other insects) undergo slow oscillations in membrane potential or alternate between firing and silence during sleep. Do they instead stop firing altogether, as is reportedly the case in some part of the mammalian brainstem? It is also unknown whether sleep and wake are accompanied by systematic changes in the levels of neuromodulators. On the other hand, since the demonstration that fruit flies sleep more than 10 years ago [36, 37], it has become especially relevant to try and identify a universal function for sleep that might apply also to invertebrates. For this reason, it seemed important to establish whether in flies, too, sleep would renormalize synapses. We now know that, at least in *Drosophila*, there is a major reduction in the number of synapses and in the expression of both pre- and postsynaptic proteins after sleep. How this renormalization happens—especially if it happens using very different mechanisms from those employed in mammalian cortex—is an intriguing question for the future.

### 4.4. On the Role of Temperature and Glucocorticoids

An important issue brought up by Frank concerns alternative mechanisms that could account for the observed changes in synaptic strength across sleep and wake: specifically, changes in brain temperature and changes in glucocorticoids levels. In principle, these mechanisms could complement the others discussed in SHY, such as the switch between tonic and burst firing that accompanies the transition from wake to sleep, and the changes in the levels of neuromodulators. As discussed below, however, the evidence supporting a role for temperature and glucocorticoids is far from compelling.

In relation to changes in brain temperature, Frank notices that the sleep/wake changes in dendritic branching and spine number that were observed in flies (e.g., a ~30% change in spine density in visual neurons) are similar to those seen in hibernators. However, during hibernation, core temperature drops by 20–30°C, and the work quoted by Frank [76] shows that there is a linear relationship between temperature and spine density: a ~30°C drop in core temperature during hibernation leads to a ~30% decrease in spine density, while a ~20°C drop results in a ~20% spine decrease. In the *Drosophila* studies, on the other hand, flies were kept inside environmental chambers whose temperature was carefully maintained at 20°C at all times. Moreover, presynaptic structural changes occurred in flies kept in small glass tubes that allow for little movement, so it is unlikely that locomotor activity could cause major changes in core temperature—of the order of 15–30°C—that are necessary to trigger massive dendritic and spine remodeling. In relation to the results obtained in rodent cerebral cortex, Frank also refers to *in vivo* studies showing that locomotor activity can enhance synaptic currents in the hippocampus by increasing hippocampal temperature by 2-3°C [77]. However, as described in detail in the original publication, molecular results (e.g., AMPA receptors changes) were obtained from rats whose cortical temperature increased by 0.3-0.4°C in wake relative to sleep [5]. Moreover, the cortical evoked responses in the two experimental conditions—“after sleep” and “after wake”—were collected in the same behavioral state, quiet wake.

Glucocorticoids can both enhance and suppress synaptic plasticity. Frank quotes evidence for their role in enhancing glutamatergic transmission and AMPA receptor trafficking, but there is strong evidence also for the opposite: stress-induced glucocorticoids also reduce synaptic efficacy in cortex [78], affect AMPA receptor trafficking in a way conducive to synaptic depression [79], and lead to long-lasting net spine elimination in cortex [80]. This last study is especially relevant because it was performed in the same mouse strain, of the same age (~1 month old), using the same method (*in vivo* repeated two-photon imaging), and focusing on the same cortical area (barrel cortex) as the study of synaptogenesis and pruning as a function of sleep and wake [9]. Maret, Faraguna, and colleagues found that spine growth and loss occur at all times, but growth prevails over loss during wake, while the opposite occurs in sleep. Liston and Gan found instead that acute and chronic corticosteroid treatment increases both spine formation and elimination, but the latter more than the former, resulting in a net decrease in spine density in the long run. Therefore, at least in the adolescent mouse cortex, it seems unlikely that the net effects of sleep/wake on spine turnover can be ascribed to glucocorticoids.

More generally, not unlike catecholamines, glucocorticoids are important for optimal performance and behavioral adaptation. Thus, it is reasonable to assume that mildly elevated levels of glucocorticoids and catecholamines during wake may both contribute to the net increase in synaptic strength observed in this behavioral state [81]. Direct evidence for this is lacking, however, and in fact the only available data suggest a very different picture. Specifically, lesion studies show that the induction of plasticity-related genes such as *BDNF* (for which a role in synaptic potentiation is overwhelming) and the associated buildup of sleep pressure are related to the activation of the noradrenergic system [59, 82, 83], while corticosterone affects neither the induction of these genes nor the homeostatic regulation of sleep [84]. Moreover, there is some evidence that low levels of catecholamines and of BDNF [32, 33, 85] may promote synaptic depression, at least *in vitro*, while no such evidence is available for low levels of glucocorticoids.

### 4.5. On the Meaning of Synaptic Homeostasis in Development

*“… sleep amounts are maximal during periods of heightened synaptogenesis including in utero when waking experience is negligible. It seems highly unlikely that a fundamental purpose of sleep is to principally weaken synapses during these developmental periods.”*

When SHY was initially proposed, it emphasized predictions that could be tested in adult mammals, where sleep would be essential in rebalancing net synaptic strength that is biased towards an increase during wake. Many of these predictions have since been corroborated. However, it was clear from the start that if sleep serves an essential function, and if that function is synaptic homeostasis, then it should apply even more to development, a time when sleep is an even more prominent part of life. Indeed, the main reason why sleep need, and thereby the need for synaptic homeostasis, would be paramount during development is fairly obvious, and it has little to do with the amount of wake: it is well known that neurodevelopment is characterized by an early phase of net synaptogenesis, followed by net pruning [4]. The increase in the number of synapses during early development is explosive, and it is bound to pose even greater challenges to neurons (and glia) than the increase in synaptic strength that occurs during wake in adult mammals: it is hard to imagine that such a massive, fast-paced formation of new synapses can be perfectly regulated, precisely titrating the total amount of synaptic weight impinging on each neuron. It is much more likely that, as a rule, during synaptogenesis neurons may undergo a substantial synaptic overload, such that proper function requires an equally substantial restoration of synaptic homeostasis. For the reasons discussed in the previous sections, such rebalancing is best achieved off-line, when a neuron can sample most of its inputs in an unbiased manner and make the necessary adjustments. The very first studies investigating the occurrence of sleep-dependent synaptic homeostasis during neurodevelopment (adolescent mice) once again support the idea that sleep is associated with a net decrease in the number of synapses [9, 13], thus possibly helping to maintain synaptic homeostasis in the face of ongoing synaptogenesis. Whether sleep plays a similar role in earlier developmental stages, the role played by different kinds of sleep, and the consequences of sleep deprivation at such critical periods on appropriate pruning and refinement of neural circuits are key questions that await investigation.

### 4.6. On “Why Stronger Synapses Should Make One Sleepy.”

Frank correctly points out that it is not clear why stronger synapses, if that is indeed the price we pay for plasticity, should produce sleepiness. It is worth remembering, however, why according to SHY renormalization is necessary: a net increase in synaptic strength comes at a substantial price to nerve cells, most relevantly in this context a price in terms of energy metabolism, since stronger synapses consume more energy [42, 43]; and in terms of cellular supplies, since stronger synapses are likely to require more building blocks and may stress the supply-and-demand requirements of neurons. After all, neurons are unique in their need to sustain thousands of synapses distributed along an extraordinary large axonal and dendritic tree, and if these synapses become on average stronger, so do their demands on the cell. There is indeed evidence that markers of cellular stress, such as BiP, are higher after wake [86]. A tendency for increased adenosine—a marker of energetic stress—has also been observed in several brain structures after extended wake [87].

Recently, it was reported that, the longer a rat stays awake, the more cortical neurons show brief periods of silence in their firing that are essentially indistinguishable from the OFF periods observed during slow oscillations in a sleeping animal [88]. These OFF periods are local, in that they may occur at different times in different brain regions, and when they occur in the wrong region at the wrong time, they can produce performance deficits. Preliminary results indicate that such OFF periods become more frequent after the induction of LTP, as well as after intense learning. While it is unknown what drives the occurrence of OFF periods at the cellular level, it is conceivable that net synaptic strengthening may have something to do with it, for example, due to increased metabolic demand. If indeed a progressively larger fraction of neurons in the brain begins to undergo local sleep, and especially if neurons in hypothalamic and brainstem areas that exert a central control on wake and arousal also suffer from synaptic overload and respond by going briefly off line, it would not be surprising if sleepiness would also increase. In fact, whether and how a net increase in synaptic strength may translate into an increased drive for hyperpolarization and an increased occurrence of OFF periods is an experimental question motivated by SHY that seems ideally suited for mechanistic investigations.

## 5. Conclusion

Frank's detailed commentary provides a helpful, critical review of the evidence concerning the mechanisms that may bring about an imbalance of synaptic homeostasis during wake and its restoration by sleep. As this response hopefully shows, it is important to distinguish between the particular mechanisms of plasticity that are engaged in the waking and sleeping brain and the universal, essential function that SHY attributes to sleep—the reestablishment of synaptic homeostasis. So far, structural, molecular, and electrophysiological studies support the notion that sleep leads to the renormalization of synaptic strength in several species. Instead, the specific mechanisms involved in the constant battle between upregulation and rebalancing of synaptic strength are bound to be many, not mutually exclusive, and different in different species, brain structures, and developmental periods. While SHY offers several corollary claims, such as the significance of sleep slow waves in mammals and birds, its core claim remains that sleep is universally needed to combat, through off-line renormalization, the neural costs of increasing synaptic strength: energy, space, cellular supplies, signal-to-noise ratios, and saturation of the ability to learn.

## Abbreviations

LTD: Long-term depression
LTP: Long-term potentiation
SHY: Synaptic homeostasis hypothesis
SWA: Slow wave activity.
